# Sport Nutrition Knowledge, Behaviors and Beliefs of High School Soccer Players

**DOI:** 10.3390/nu9040350

**Published:** 2017-04-01

**Authors:** Melinda M. Manore, Megan M. Patton-Lopez, Yu Meng, Siew Sun Wong

**Affiliations:** 1 School of Biological and Population Health Sciences, College of Public Health and Human Sciences, Oregon State University, Corvallis, OR 98331, USA; mengy@oregonstate.edu; 2 Family and Community Health, School of Biological and Population Health Science, College of Public Health and Human Sciences Oregon State University, Corvallis, OR 98331, USA; Megan.Patton-Lopez@oregonstate.edu (M.M.P.-L.); SiewSun.Wong@oregonstate.edu (S.S.W.)

**Keywords:** Latino, free or reduced lunch, supplement use, low-income, National School Lunch Program, adolescent athletes, diet behaviors

## Abstract

For adolescent athletes (14–18 years), data on sport nutrition knowledge, behaviors and beliefs are limited, especially based on sex, race/ethnicity and socioeconomic status. High school soccer players (*n* = 535; 55% female; 51% White, 41% Latino; 41% National School Lunch Program (NSLP) participants (80% Latino)) completed two questionnaires (demographic/health history and sport nutrition). The sport nutrition knowledge score was 45.6% with higher scores in NSLP-Whites vs. NSLP-Latinos (*p* < 0.01). Supplement knowledge differed by sex (16% lower in females; *p* = 0.047) and race/ethnicity (33% lower in Latinos; *p* < 0.001). Breakfast consumption was 57%; females ate breakfast less (50%) than males (60%; *p* < 0.001); NSLP-participants ate breakfast less (47%) than non-NSLP (62%; *p* < 0.001). Supplement use was 46%, with Latinos using more supplements than Whites do (*p* = 0.016). Overall, 30% used protein shakes, with females using less than males (*p* = 0.02), while use was twice as likely in Latino vs. White (*p* = 0.03). Overall, 45% reported their nutrient requirements were different from non-athlete peers. Latinos were less likely (*p* = 0.03) to report that their diet met nutritional requirements, but more than twice as likely to report that nutritional supplements were necessary for training (*p* < 0.001). Adolescent athletes, especially females and Latinos, would benefit from sport nutrition education that enhances food selection skills for health and sport performance.

## 1. Introduction

As adolescents age, physical activity decreases [1,2,3], while the independence to make food choices increases [4]. The diet and physical activity behaviors learned in these early years can continue into adulthood, thus, impacting long-term health and weight [4,5]. Without positive reinforcement to make good food choices and maintain or increase physical activity, the risk of being overweight and/or obesity increases as adolescents transition into adulthood [4]. Thus, capturing adolescents while they are still active and engaged in youth sports may be a “window of opportunity” to cultivate skills that support life-long health and obesity prevention, such as healthy eating behaviors, grocery shopping and cooking skills, and reinforcing the importance of daily physical activity. This is also an opportunity to teach youth athletes how to fuel and hydrate their body for physical activity and to discern if sport foods and supplements are needed. Unfortunately, adolescent athletes do not always make healthful food choices or have the best food options available to them at sporting events. Based on a review by Nelson et al. [6] only seven studies have compared the dietary intake of adolescents engaged in sports to non-sport participants. They found that adolescents involved in youth sports were more likely to consume fruits, vegetables and milk, but were also more likely to eat fast food and consume sugar-sweetened drinks than non-participants. In addition, parents report that few healthful foods and beverages are available to adolescent athletes at sporting events, thus, increasing the consumption of these foods [7]. If adolescent athletes can learn how to fuel their body for sport by selecting healthy foods and appropriate beverages, they may establish and carry these diet behaviors into adulthood [8]. Finally, while adolescent athletes are still engaging in high school sports, this is an opportunity to teach them about the benefits of life-long physical activity.

Most research using adolescent athletes (14–18 years) report energy and nutrient intakes, while limited research has examined sport nutrition knowledge, behaviors and beliefs. Studies that have examined these topics in adolescent athletes have focused on elite or club level athletes [9,10,11,12,13], while only two have examined high-school level athletes [13,14,15,16]. Perron and Endres [14] examined both nutrition knowledge and attitudes in high school female volleyball players and found that higher nutrition knowledge was positively correlated with a more positive attitude toward nutrition, but did not translate into better food choices. Walsh et al. [15] reported that their male high school rugby players demonstrated poor nutrition knowledge of the foods needed for fueling during and after sport (60%), while alcohol and dietary supplement use was high at 87.7% and 64.5%, respectively.

None of the above studies have examined differences in sport nutrition knowledge, attitudes and beliefs in adolescent athletes based on sex, race/ethnicity, or socioeconomic status. Female adolescent athletes have different nutrition and body weight and image issues compared to adolescent male athletes [17,18]. They are also at greater risk for health issues related to poor nutrient and energy intakes, such as iron deficiency anemia, disordered eating, stress fractures, and the female athlete triad [19,20,21].

Soccer is the world’s most popular sport, including the Latino population within the United States (US) [22], yet little is known about the sport nutrition behaviors and beliefs of adolescent US Latino athletes. In addition, participation of adolescent Latina girls in organized sport is lower than their peers [23], yet findings from the National Health and Nutrition Examination Survey (NHANES) 1999–2006 suggest that Latinas are more likely to participate in soccer compared to White or Black females [24]. To date no research has examined the sport nutrition knowledge, attitudes and beliefs of adolescent Latino athletes.

Finally, the socioeconomic status and level of food insecurity of an adolescent athlete’s family can impact food choices available in the home [25] and thus, the energy and nutrient intake of the adolescent athletes. To our knowledge, no study has considered the economic status of the athlete’s family when examining their nutritional status, or their sport nutrition knowledge, behaviors or beliefs.

Thus, the purpose of this study was to examine the sport nutrition knowledge, behaviors, and beliefs of high school soccer players and determine if there were differences based on sex, race/ethnicity (Latino, White), and socioeconomic status.

## 2. Materials and Methods

### 2.1. Experimental Design, Participants and Recruitment

High school soccer players (*n* = 535; 14–18 years; 13 schools and 24 soccer teams) were recruited for a 2-year integrated (research, education and Extension) sport nutrition and life-skills intervention (The WAVE~Ripples for Change: Obesity Prevention in Active Youth). Goals and details of the larger study can be found at the USDA web site [26]. Overall, the participants were 55% female, 41.3% in grades 11 and 12, predominately White (51%) and Latino (41%), and reported playing soccer an average of 7.5 years. As a group, 40.6% participated in National School Lunch Program (NSLP) and 59% reported no sport injuries during the last 12-months. This manuscript focuses on baseline data examining the sport nutrition knowledge, behaviors, and beliefs of these soccer players before beginning the intervention. All questionnaires used were pilot tested with male and female high school soccer players (*n* = 53) and appropriate changes made before administration to participants. Athletes were recruited through their soccer coaches from high schools located in the Willamette Valley, Oregon. The University Institutional Review Board approved the study (Approval No. 6317) and all participants and their parents/guardians were informed prior to signed assent/consent.

### 2.2. Baseline Assessments and Questionnaires

All baseline assessments and questionnaires were completed at the participating schools. Height (m) was measured using a free-standing stadiometer (SECA 217, SECA Corp., Singapore), where subjects stood upright in bare feet with heals together, looking straight ahead. Weight (kg) was measured using a calibrated Tanita body-composition analyzer (TM-300A, Tanita Corp., Itabashi-Ku, Tokyo, Japan). Participants were measured prior to any strenuous physical activity in bare feet, with light weight soccer clothing, after a 4 h fast from food and with an empty bladder. Body composition data are not reported because the majority of participants were younger than 17 years and there were no validated equations for athletic mode on the Tanita scale for this age group. Overall, 618 youth provided assent/parental consent to participate in the study; 535 completed all the necessary questionnaires and had height and weight measured (86.6% response rate).

All participants completed two questionnaires: (1) a demographic and health history questionnaire designed for the study that included questions regarding the number of years playing soccer, sport related injuries, and general eating patterns and (2) a validated sport nutrition questionnaire [15]. Participants also indicated their participation in the NSLP, which was used as a proxy for low household income [27]. The sport nutrition questionnaire focused on eating practices and behaviors around sport, attitudes and beliefs about food and nutrition relative to sport performance, and sport nutrition knowledge and supplement use (vitamins/minerals, herbal products, protein shake/supplement). The questionnaire had been previously validated in high school rugby players in Ireland by Walsh et al. [15]. The questionnaire consisted of 40 questions, including questions regarding training schedule. Only minor changes were made to the questionnaire to include foods typically consumed in the US (e.g., biscuits to cookies, crisps to potato chips, chips to French fries) and references to rugby were replaced with soccer (e.g., ‘As a rugby player’ to ‘As a soccer player’). Table 1 outlines the key themes and topic areas addressed in the sport nutrition questionnaire. The complete questionnaire is available from Walsh et al. [15]. A sport nutrition knowledge score was calculated for each athlete by adding the total number of correct questions (*n* = 12) covering four domains (hydration, protein/carbohydrate, supplements, and pre/post exercise food selection). The sport nutrition questionnaire was administered in the presence of the researchers to minimize discussion of responses between participants, and no members of the coaching staff were present. Cronbach’s alpha was used to estimate internal reliability for the nutrition knowledge subsection, which was determined to be 0.51.

### 2.3. Statistical Analysis

**Figure 1:**
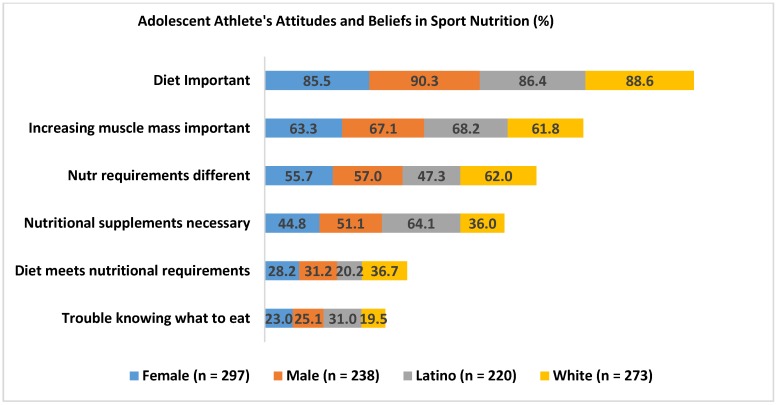
Four major sources of sport nutrition information (% reporting each source) for high school soccer players by participation in the National School Lunch Program (NSLP) and race/ethnicity (Latino, White) (*n* = 535). There were no statistical differences between groups. All = all participants; NSLP = National School Lunch Program; Non-NSLP = those not participating in NSLP; Race/ethnicity = Participants self-identified as White or Latino.

Descriptive statistics (mean, standard deviation (SD)) were calculated to summarize the baseline characteristics of the sample. Independent *t*-tests were used to examine differences between males and females for age, years playing soccer, and age that participant began preparing meals for themselves. Chi-squared tests were used to examine differences between males and females for discrete variables (race/ethnicity, year in school, no injuries in the past 12 months, participation in the NSLP, and prepares meals for self). Chi-squared tests were also used to compare source of sport nutrition information between subgroups based on race/ethnicity and NSLP-participation (variables in Figure 1). Multivariable Poisson regression was used to examine differences in sport nutrition knowledge based on sex, race/ethnicity (White, Latino, Other) and participation in the NSLP (yes, no) and their interaction effects, and total knowledge score and specific knowledge domain scores (hydration, carbohydrate/protein knowledge, supplement knowledge, and pre/post exercise food selection). Multivariable logistic regression was used to examine differences in sport nutrition behaviors (eat breakfast daily, eating within 1 h before/after training/games, supplement use, and protein shakes/meal replacement use) and attitudes/beliefs (importance of diet, muscle mass, supplements, adequacy of current diet, nutritional requirements compared to peers, and food selection) based on gender, race/ethnicity, and income and their interaction effects. All results were adjusted for year in school, years playing soccer, and preparing meals for themselves. Associations are quantified by relative means (RM) and odds ratios (OR) with 95% confidence intervals (CI). Likelihood ratio (LR) tests were used for model selection (e.g., to compare models with and without interaction terms). Adjustment for multiple comparisons was made by using Hochberg’s method [28]. Both unadjusted and adjusted-for-multiple comparisons *p*-values are presented. All analyses were conducted using Stata SE 14.2 (College Station, TX, USA).

## 3. Results

### 3.1. Demographic Characteristics

Demographic characteristics of the group are presented in Table 2. Participation in the NSLP was 40.6%, with more males (46.2%) than females (36%) (*p* = 0.020). Overall, 80% of NSLP participants were Latino and 17% were White. Females reported preparing their own meals earlier (age 11) than males (age 12) (*p* < 0.001).

### 3.2. Sport Nutrition Knowledge and Sources of Nutrition Information

Sport nutrition knowledge results are presented in Table 3. The mean number of sport nutrition knowledge questions answered correctly was 5.5 ± 2.0 or 45.6%. Only 6% of the group had a sport nutrition knowledge score of 75% or greater. Using Poisson regression there were no main effects in sport nutrition knowledge for sex (*p* = 0.080), but interactive effects for race/ethnicity and NSLP participation (*p* < 0.017). For NSLP participants, Whites scored significantly higher (23%) than Latino (*p* = 0.01 unadjusted, *p* = 0.048 adjusted; 95% CI = 6%–44%). Using the same analyses, the four sport nutrition knowledge domains showed a significant main effect for supplement knowledge, but no interactive effects for sex, race/ethnicity or NSLP participation. For supplement knowledge, Latinos had a significantly lower score than Whites (33% lower; *p* = 0.004). Overall, the highest average sport nutrition knowledge scores were in hydration (≥76% correct), followed by supplementation knowledge (52% correct), protein/carbohydrate knowledge (35.5% correct) and pre/post exercise food selection (24.3% correct).

Figure 1 shows where high school soccer players sought sport nutrition information. Overall, only 24% indicated they had sought dietary advice for sport, and of these the top four sources were family or medical professional (12.7%), coach/trainer (12%), internet (10.3%) or friends and teammates (8.8%). Where participants sought sport nutrition information was not different base on race/ethnicity or NSLP participation.

### 3.3. Dietary Behavaiors Relevant to Sport Performance

Overall, 55.7% of participants reported eating breakfast daily, 36.6% reported eating 1-h before training/games, and 79.4% reported eating within 1-h following training/games. Supplement use was reported by 46.4% of participants and 30.1% reported using a protein shake or meal replacement beverage.

Multivariate logistic regression was used to examine differences in dietary behaviors based on main effects (sex, race/ethnicity, and NSLP participant) (see Table 4). There were no interactive effects for dietary behaviors, thus, only main effects were examined. For each diet behavior question, participants responded either ‘yes/no’ to participating in the behavior or selected an option that best reflected their behavior. For eating breakfast daily, there was a main effect for sex, with females (49.8%) less likely to report eating breakfast daily than males (63.0%) (OR = 0.50; CI = 0.34, 0.73). There was also a main effect for NSLP participation, with NSLP participants less likely to eat breakfast daily (47.0%) than non-NSLP participants (61.7%) (OR = 0.40; CI = 0.24, 0.67). For eating within 1-h after training/games there was a main effect for sex, with females more likely to eat within 1-h (84.2%) than males (73.5%) (OR = 1.93; CI = 1.23, 3.04). For general supplement use, there was a main effect for race/ethnicity with Latino participants being more likely to report regular supplement (56.6%) use compared to Whites (39.3%) (OR = 2.07; CI = 1.30, 3.40). For use of protein shakes or meal replacement beverages, there were main effects for sex and race/ethnicity (*p* = 0.02); however, when adjusted for multiple comparisons there were no differences (*p* = 0.07) (see Table 4). Overall, males typically used protein shakes/beverages more frequently (36.1%) than females (25.2%) and Latinos used protein shakes/beverages more frequently (40.2%) than Whites (22.0%). There were no main effects for eating 1-h before training/games. Water was the preferred beverage of choice before (97%), during (80.6%) and after (94%) exercise. Sport drink consumption was 47.3% before exercise, but use dropped to 12.0% and 14.7% during and after exercise, respectively. Fruit juice/drinks and diluted sport beverages were also consumed before (21.4%), during (5.8%), and after (36.3%) exercise, while few selected milk (7.3%) as a post-exercise beverage.

### 3.4. Attitudes and Beliefs Relevant to Sport Nutrition

**Figure 2:**
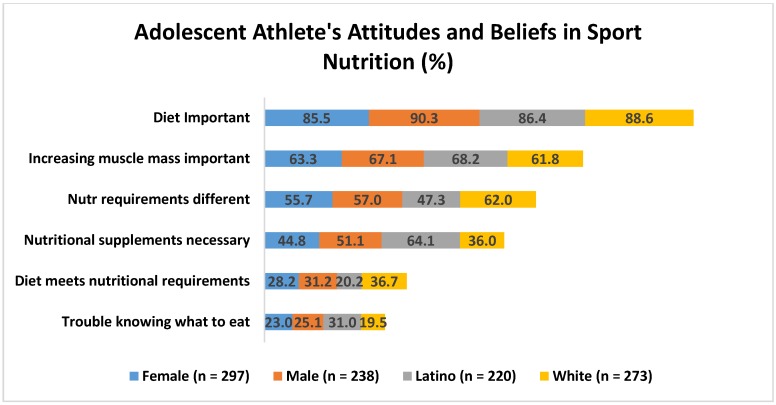
Self-reported attitudes and beliefs relevant to sport performance by sex and race/ethnicity (White, Latino). Percentage of participants from each category indicating this statement was true. Race/ethnicity = Participants self-identified as White or Latino.

Figure 2 graphically shows the attitudes and beliefs relevant to sport performance between groups (see Table 5 for statistical analysis of these data). Only 20.2% of Latino participants indicated that they felt their diet/eating plan met nutrition requirements, compared to 36.7% of White participants. More Latino participants (64.1%) reported that nutrition supplements were necessary to support training program vs. Whites (36.0%). Similarly, more Latino participants (31%) than Whites (19%) reported they had trouble knowing what to eat for sport. Overall, 45% of athletes reported that their nutrient needs were different than that of other people their age.

Multivariate logistic regression was used to examination differences in dietary attitudes and beliefs relative to sport performance based on sex, race/ethnicity, and participation in the NSLP (see Table 5 and Figure 2). Overall, there were no differences based on NSLP participation. Both male and female participants reported that diet was important for performance 90.3% and 85.5%, respectively. There was a significant sex by race/ethnicity interaction (*p* < 0.02) for ‘the belief that nutrition requirements are different for athletes compared to peers’; however, after correcting for multiple comparisons there were no differences (*p* > 0.10) (see Table 5). Overall, 63.6% of White and 37.5% of Latina females indicated their nutritional needs were different than that of their peers, compared to similar values in males (White = 59%; Latino = 56%). Only 29.5% of youth reported that their diet met their nutritional requires. There was a significant main effect for race/ethnicity (*p* = 0.028) for this question, but no differences once adjusted for multiple comparisons (*p* = 0.11) (see Table 5). Nearly 37% of White participants agreed that their diet met their nutritional requirements compared to 20.2% of Latino participants. Finally, Latino participants were 2.4 times more likely to report that nutritional supplements were necessary to support their training program (OR = 2.43; CI = 1.48–3.97; *p* < 0.001) compared to their White peers. Overall, 47.6% of youth believed that nutritional supplements were necessary to support their training.

## 4. Discussion

This is the first study to determine whether the sport nutrition knowledge, behaviors, and beliefs of high school soccer players differ based on sex, race/ethnicity, or socioeconomic status. The most significant finding of this study was that high school soccer players had sport nutrition knowledge scores (45.6%) lower than those reported in the research literature for adolescent athletes (typically >65%) [9,13,14], with scores lowest among NSLP participants who were Latino (38.8%). In addition, girls and those participating in the NSLP were less likely to eat breakfast. Overall, eating behaviors around sport were similar among all three race/ethnic groups; however, Latino males were twice as likely to use supplements than their White counterparts. Dietary beliefs and attitudes were also similar among groups except for supplementation beliefs in Latino participants, who reported that nutritional supplements were needed to support their training. Overall, 47%–62% of youth, depending on the group, thought their diets were different from their non-athletic peers and only 20%–35% of youth thought their current diets met their nutritional needs.

### 4.1. Sport Nutrition Knowledge and Sources of Nutrition Information

Assessment of general nutrition or sport nutrition knowledge is limited in high school age athletes (13–18 years), especially those not participating at elite or club levels [14,15]. There are no studies specifically examining sport nutrition knowledge in high school soccer players. Overall, mean general nutrition or sport nutrition knowledge scores for high school athletes range from 55%–74% [9,10,11,12,13,14,15], with higher scores in females (65%–68%) [12,13,14]. Our results (45.6%) are lower than those reported by others [9,10,11,12,13,14], but are most similar to Walsh et al. [15] and Spronk et al. [11]. Walsh et al. examined sport nutrition knowledge in Irish senior male high school rugby players (59.6% overall score), while Spronk et al. [11] reported general nutrition knowledge scores of 55% in elite male athletes (≥16 years). Although our questionnaire was adapted from Walsh et al., our scores were still 15% lower. In addition, the athletes in these two studies [11,15] were primarily male, played rugby, and were more elite or older than participants in the present study. Another reason our results might be lower than those reported in the research literature is that there is no standardized sport nutrition questionnaire used by researchers. Many researchers use ‘general nutrition questionnaires’ [10,11,12,13], while others use general nutrition questionnaires with some sport nutrition questions added [9,14,16]. High school students may do better on general nutrition questionnaires, especially if they have had health or life-skills class at school. Sport nutrition is more specific and may not be addressed in these classes.

The lower sport nutrition knowledge scores observed in this study, compared to the research literature ([9,10,11,12,13,14,16]) may be attributed to three factors: (1) Participants were high school athletes who were younger and less elite; (2) Participants were ethnically and economically diverse; and (3) Participants had limited access to knowledgeable sources of sport nutrition information. Each of these factors are discussed below in more detail.

Firstly, younger athletes who are less elite may have less exposure to nutrition education/sport nutrition information or have less interest since they are still learning the skills of their sport. Most researchers examining nutrition knowledge in youth athletes use elite or club level athletes. Our participants were young (mean age = 15 years; 58% 9th and 10th grades) and not elite/club level athletes. Spendlove et al. [10] found that in their elite female athletes (mean age 18.9 years) scored higher in general nutrition knowledge than male athletes. Finally, Little et al. [29] found that freshman and sophomore non-athlete high school students scored significantly lower on general nutrition knowledge tests than juniors and seniors.

Secondly, few have examined sport nutrition knowledge in an ethnically diverse group of young athletes. Only Spendlove et al. [10] has examined differences in race/ethnicity and general nutrition knowledge in athletes, but their athletes were more elite and older (mean age = 18.9 years) than the athletes in our study. They also examined general nutrition knowledge and not sport nutrition knowledge. Their results showed no differences in general nutrition knowledge based on race/ethnicity, but their sample was primarily (96.6%) Western (Australian, United Kingdom or North America) athletes. Thus, there was little variability in race/ethnicity. Overall, 41% of our participants were Latino, with the majority participating in the NSLP (80%). These Latino athletes had significantly lower sport nutrition knowledge scores than their White peers. The sport nutrition knowledge questionnaire used was validated using high school elite rugby players from Ireland [15]. We adapted the questionnaire for US foods, but did not test for culture appropriateness with Latinos. Soccer is the most popular sport in the Latino culture, and 56% of Americans who identify as Hispanic or Latino follow the sport [22]. Thus, Latinos are very familiar with the sport, but may not be familiar with the sport nutrition recommendations for team sports such as soccer [8,30,31]. Finally, Little et al. [29] reported low general nutrition knowledge scores (~36%) in high school students (72% African-American; 10% Hispanic) identified as NSLP participants. Neumark-Sztainer et al. [32] also reported that adolescents from low socioeconomic backgrounds were at greater risk for inadequate food intake patterns. Thus, low-income high school students may not have the knowledge, familiarity or resources to make better food choices.

Finally, 30% of our participants reported seeking sport nutrition information. Their primary sources of information were coaches and family/medical professionals (~12% each), internet (10%) and friends/peers (8%). None of these are optimal sources of sport nutrition information. A comprehensive review of coaches nutrition knowledge by Trakman et al. [33] indicated that key sport nutrition concepts are poorly understood by most coaches. In addition, coaches who do the job full-time or coach higher level athletes may be more interested in providing accurate sport nutrition [34] to their athletes compared to part-time coaches. These data suggest that most high school coaches are not trained to give appropriate sport nutrition information, yet athletes rely on them for this information. At higher levels of competition, such as national or Olympic level sports, many athletes have access to sport dietitians.

### 4.2. Dietary Behaviors Relevant to Sport Performance

Limited research has examined the diet behaviors of young athletes (≤18 years) and no consistency in the items examined were found; thus, comparisons to others is difficult. We found that 56% of our participants reported eating breakfast most days of the week, while Tawfik et al. [16] reported that 79% of their young Egyptian athletes (mean age: 14.3 years, *n* = 358) consumed breakfast. Croll et al. [35] found that adolescents involved in sport (*n* = 1715) had higher rates of eating breakfast (males = 4.7 times/week; females = 3.6 times/week) than those not involved with sport (males = 3.7 times/week; females = 3.2 times/week) (*n* = 838). Similar to others [16,35], we found that our male athletes (63%) ate breakfast more frequently than our females athletes (50%). Athletes participating in the NSLP ate breakfast less frequently (49.8%) than non-NSLP participants (61.7%), even though the free or reduced breakfast was offered in all schools. Thus, active, growing youth who skip breakfast eliminate a nutrient dense, high quality meal from their diet that can boost total daily nutrient intake [36,37,38].

The consumption of food before a sport practice/game in our participants was similar (36.6%) to that reported by Walsh et al. [15] in high school rugby players (26%). Conversely, a majority of our athletes (>74%) consumed food within 1 h after exercise. Walsh et al. [15] reported that 62% of their athletes consumed food within one-half hour after exercise, while Nascimento et al. [9] reported that only 40% of their adolescent athletes (*n* = 11; 12–19 years) had adequate pre- and post-exercise meals at baseline. For our high school athletes, sport practice/games occurred right after school, thus, the ability to eat 1–2 h before exercise may not have been possible, depending on the availability of food and the ability to eat in the classroom.

For the adolescent athletes in our study, 46% reported supplement use of some type (multivitamin/ mineral, herbal, protein), with the most common supplement being protein shakes/meal replacements (30%). Others report supplement use in adolescent athletes [15,39,40] to range from 17% to 65%. We also found male athletes use protein shakes/meal replacement products more (36%) than female athletes (25%), and Latino athletes (40%) more than White athletes (22%). Only two other studies have examined racial/ethnic differences in supplement use among adolescents. George and colleagues [41] examined associations among dietary supplement use and dietary/activity patterns in a diverse sample of Texas 11th graders (non-athletes). They found that the highest supplement use among White youth compared to Mexican-American and Non-Hispanic Black youth. Similarly, Evans et al. [39] also examined dietary supplement use in children and adolescents participating in the 2007 National Health Interview Survey. They found that 60% of White youth reported usage compared to 20% of Blacks and 12.5% of Hispanics. However, these studies were not separating participants based on sport participation. We found no differences in supplement use between athletes participating in NSLP (e.g., low-income) and non-NSLP participants after controlling for key variables.

Protein supplements are popular with active individuals, especial male athletes. We found that 30% of our participants were regularly using protein beverage/shakes. Two other studies have examined protein supplement use in high school athletes and report use at 18%–43% [15,42]. Similar to Parnell et al. [42] we found that females were less likely to use protein beverages/shakes compared to males. When protein supplement use was examined based on race/ethnicity, Latino youth were almost twice as likely to supplement (OR = 1.84, CI = 1.08–3.13). To our knowledge, no other study has examined racial/ethnic differences in protein supplement usage among adolescent athletes. However, data from Eisenberg and colleagues [43] suggest that within a diverse group of adolescents (mean age = 14.4 years), Latinos (32%) use more protein powder/shake compared to Whites (25.3%).

### 4.3. Dietary Beliefs Relevant to Sport Performance

Dietary beliefs about sport nutrition may impact an athlete’s food and supplement choices. One unique finding of this study was that among our group of adolescent athletes, Latinos were twice as likely to believe that nutrition supplements were necessary to support training program compared to Whites. The belief that sport nutrition supplements will enhance performance use is not unusual in athletes [44], but research has not examined this trend in adolescent Latino athletes living in the US. One possible explanation for higher use in Latino athletes may be related to the higher use of supplements by Hispanics adults. Based on a review of the 2011 Gallup Study of Hispanic Nutrition and Supplement Use, 74% of adult Hispanics use supplements and 18% of Hispanic men between the ages of 18–24 use sport supplements [45]. In addition, Hispanics are more likely to buy nutritional products for their children compared to other demographics [46]. These factors may all contribute to why our Latino athletes had higher supplement use.

### 4.4. Strengths and Limitations

Our study had a number of strengths. Firstly, we had a large sample (*n* = 535) of high school soccer players, with an average of 7 years’ experience playing the sport; thus, they may be representative of many high school level athletes. Participants completed questionnaires in the absence of coaching staff. Secondly, we had nearly equal numbers of male and female (55%) athletes and a high percentage (41%) of Latino participants. Thirdly, 41% of our athletes were NSLP participants, an indicator of low socioeconomic status. Thus, we had a diverse enough sample to examine the impact of sex, race/ethnicity, and socioeconomic status on sport nutrition knowledge, behaviors, and beliefs in these high school athletes. Finally, we used a sport nutrition questionnaire that had been validated in high school athletes playing a team sport. The use of non-validated questionnaires for general or sport nutrition knowledge has been identified as a weakness in this area of research [47].

There were also limitations to this study. Firstly, although the sport nutrition questionnaire was validated [15] and revised with typical US sport and snack foods, we did not test for cultural appropriateness. We also do not know the acculturation level of our athletes. Secondly, many of the questions were restricted to yes/no responses or had limited options to select from, which did not allow participants to provide more detailed information about their dietary behaviors. Other questions allowed for graded responses and these responses were collapsed for some questions into fewer groups for reporting purposes. Thirdly, we did not test the sport nutrition knowledge of the coaches or ask what sport nutrition recommendations they provided to their athletes. Many of the soccer coaches were volunteers who played soccer. Knowing this information would have helped us better understand our sport nutrition knowledge scores and why supplement use was high.

## 5. Conclusions

Few high school athletes become college or professional athletes, yet their attitudes and beliefs about diet, food, supplements and physical activity could influence their health and weight for a lifetime. One benefit of teaching sport nutrition and diet skill building with adolescent athletes is that sport participation can help increase their interest in food and nutrition, especially in adolescent males who are notoriously hard to reach. High school athletes already know how to be active and fit so they can perform well, thus, half the battle is won. Adding healthy food selection, cooking and nutrition to their skill set, can provide a foundation for a lifetime of good health. It is especially important to reach females and ethnic minorities. Female athletes typically have a high concern for body weight and need food and nutrition guidance to fuel their sport and avoid unnecessary dieting and body image issues. Ethnic minorities, especially Latinos, need appropriate sport nutrition recommendations that support cultural practices, yet provide the energy and nutrients needed to play well. Finally, we need to understand why active youth report their diet does not meet their nutritional needs for sport, and why active Latino youth turn to supplements over food.

## Figures and Tables

**Table 1:** Key themes covered in the sport nutrition questionnaire ^a^.

Theme	Topics Addressed by the Questions
**Training Schedule**	Position played, training schedule, hours of training during and outside school.
**Eating practices and behaviors around sport**	Typical eating patterns (breakfast, lunch, dinner, snacks); types of fluids consumed; timing of food/beverage intake before/after exercise; typical foods consumed.
**Attitudes and beliefs about food/nutrition relative to sport**	Importance of food/beverages consumed for sport performance; statements about diet that apply to them; nutritional needs of athletes are different from non-athlete peers.
**Sport Nutrition Knowledge**	**Hydration**—timing of sport drink consumption; impact of dehydration on performance. **Pre/Post Exercise Food Selection**—foods consumed following sport training or game. Best food options 3–4 h prior to sport training and games and 1–2 h post-exercise training or game. **Protein/Carbohydrate Knowledge**—Timing and amount of carbohydrate and protein consumption around sport. Consequences if amounts are low; benefits if amounts are adequate. **Supplement Knowledge**—Accuracy of supplement claims regarding benefits; Need for nutritional supplements.

^a^ The complete questionnaire can be found at Walsh et al. [15].

**Table 2:** Characteristics of female and male high school soccer players.

Demographic Characteristics	Total Sample (*n* = 535)	Female (*n* = 297)	Male (*n* = 238)
	**Mean (SD)**		
**Height (cm)**		162.6 (6.7)	172.8 (7.8)
**Weight (kg) ^a^**		59.5 (10.4)	65.0 (12.2)
**Body Mass Index (kg/m^2^)**		22.5 (3.5)	21.7 (3.4)
**Age (year)**	15.3 (1.14)	15.2 (1.1)	15.4 (1.2)
**Age preparing meals for self (year)**	11.4 (2.2)	11.0 (2.3)	12.0 (2.0)*
**Years playing soccer**	7.5 (3.4)	7.4 (3.7)	7.6 (3.8)
	***n*** **size (%)**		
Race/Ethnicity *			
Latino	220 (41.1)	104 (35.0)	116 (48.7)
White	273 (51.0)	174(58.6)	99 (41.6)
Other ^b^	42 (7.9)	19 (6.4)	23 (9.7)
**Year in School**			
9th grade	174 (32.5)	102 (34.3)	72 (30.3)
10th grade	138 (25.8)	83 (27.9)	55 (23.1)
11th grade	125 (23.4)	63 (21.2)	62 (26.1)
12th grade	96 (17.9)	48 (16.2)	48 (20.2)
**No injuries past 12-months**	316 (59.1)	178 (59.9)	138 (58.0)
**Participate in NSLP ^c^**	217 (40.6)	107(36.0)	110 (46.2) *
**Latino NSLP**	174 (80.2)	77 (74.8)	97 (83.6)
**White NSLP**	37 (17.1)	25 (14.4)	12 (12.1)
**Other NSLP**	6 (2.8)	5 (26.3)	1 (4.4)
**Prepares meals for self (%)**	56.1	57.9	53.8

* Groups are significantly different (<0.02) using *t*-test for variables reported in means and chi-squared tests for categorical variables, confidence at 95%. ^a^ Mean weight based on *n* = 284 due to missing data; ^b^ Other = African American, Asian-Pacific Islander, American Indian/Alaska Native; ^c^ NSLP = National School Lunch Program participation is used as an indicator of socioeconomic status.

**Table 3:** Sport nutrition knowledge total score by race/ethnicity, sex and participation in National School Lunch Program (NSLP) (*n* = 535). ^a^

Variable	Female (*n* = 297)		Male (*n* = 238)	
Sport Nutrition Knowledge Total Score	% ^b^	Mean (SD)	%	Mean (SD)
Average	45.1	5.4 (2.1)	46.2	5.5 (2.0)
White	48.4	5.8 (1.9)	52.1	6.3 (1.8)
Latino	38.8	4.7 (2.0)	41.6	5.0 (2.0)
Other ^c^	49.1	5.9 (2.2)	43.8	5.3 (2.3)
	**NSLP participant (*n* = 217)**		**Non-NSLP participant (*n* = 316)**	
Sport Nutrition Knowledge Total Score	%	Mean (SD)	%	Mean (SD)
Average	41.1	4.9 (2.1)	48.6	5.8 (1.8)
White	49.8	6.0 (0.1)	49.8	6.0 (1.8)
Latino	38.8	4.7 (2.0)	45.9	5.5 (1.6)
Other	54.2	6.5 (2.3)	44.9	5.4 (2.2)

^a^ Data adjusted for grade in schools, number of years playing soccer and preparing meals for oneself; ^b^ Percentage is based on 12 possible points; ^c^ Other includes participants who self-identified as American Indian/Alaska Native, Asian/Pacific Islander, or Black/African American.

**Table 4:** Multivariable logistic regression of diet behaviors related to sport nutrition by sex, race/ethnicity and participation in the National School Lunch Program (NSLP) (*n* = 535). ^a^

Variable	Odds Ratio (OR) ^b^	95% Confidence Interval (CI)	Unadjusted *p*-Value	Adjusted *p*-Value
**Eat breakfast daily**				
Female	0.50	0.34–0.73	<0.001	<0.001 *
NSLP	0.40	0.24–0.67	<0.001	<0.001 *
**Eat within 1-h before training/game**	No differences observed for groups			
**Eat within 1-h after training/game**				
Female	1.93	1.23–3.04	0.004	0.016 *
**General Supplement Use**				
Latino ^c^	2.07	1.3–3.4	0.004	0.016 *
**Use of protein beverage/shakes**				
Females	0.63	0.42–0.93	0.021	0.075
Latino ^c^	1.84	1.08–3.13	0.025	0.075

* There were no interactive effects, so only significant main effects are listed under each variable ^a^ Models adjusted for school grade (9–12), years playing soccer, and whether participants preparing meals for self; ^b^ An OR less than 1.0, indicates that participants were less likely to engage in a behavior, while an OR greater than 1.0, indicates they were more likely to engage in the behavior; ^c^ Race/Ethnicity = Participants self-identified as White, Latino or Other (American Indian/Alaska Native, Asian/Pacific Islander, Black/African American).

**Table 5:** Multivariable logistic regression estimates of dietary beliefs and attitudes relevant to sport performance by sex, race/ethnicity and participation in National School Lunch Program (NSLP) (*n* = 535). Model was adjusted for school grade (9–12), years playing soccer, and whether participants preparing meals for self. Only significant main or interactive effects are listed under each variable.

Variable	Odds Ratio (OR) ^a^	95% Confidence Interval (CI)	Unadjusted *p*-Value	Adjusted *p*-Value
**Diet is important to sport performance**				
Female	0.57	0.32–0.99	0.049	0.196
**My nutrition requirements different compared to peers**				
Female White ^b^	2.04	1.10–3.77	0.023	0.115
Latino Males ^c^	2.03	1.13–3.66	0.018	0.108
**I have trouble knowing what to eat for sport**	No differences observed for any group			
**My diet meets my nutritional requirements**				
Latino	0.53	0.30–0.93	0.028	0.112
**Increasing muscle mass is important to my performance**	No differences observed for any group			
**Nutrition supplements are necessary for training**				
Latino	2.43	1.48–3.97	<0.001	<0.001 *

* Significant difference between Latino and other race/ethnic groups (White and Other). ^a^ An Odds Ratio (OR) less than 1.0, indicates being less likely to respond ‘yes’ or rank high in importance compared to the reference group, while an OR greater than 1.0, indicates the opposite; ^b^ Comparison group is Latina Females; ^c^ Comparison group is Latina Females.
